# Copyright and Digital Teaching Exceptions in the EU: Legislative Developments and Implementation Models of Art. 5 CDSM Directive

**DOI:** 10.1007/s40319-022-01179-6

**Published:** 2022-04-05

**Authors:** Giulia Priora, Bernd Justin Jütte, Péter Mezei

**Affiliations:** 1 Assistant Professor in Private Law, NOVA School of Law, Lisbon, Portugal; 2 Assistant Professor in Intellectual Property Law, Sutherland School of Law, University College Dublin, Dublin, Ireland; 3 Associate Professor, Faculty of Law and Political Sciences, University of Szeged, Szeged, Hungary

**Keywords:** EU copyright law, Exceptions and limitations, Digital teaching activities, Digital single market, Fair balance

## Abstract

Article 5 of the 2019 EU Directive on Copyright in the Digital Single Market (CDSM) attempted to modernize the regime of copyright exceptions and limitations related to teaching activities. Its aim is to enhance the flexibility behind permitted uses to the benefit of educational institutions regarding their digital and cross-border teaching. The pressing need for such a legislative reform was confirmed by the outbreak of the COVID-19 pandemic, which dramatically moved teaching environments to online platforms. This paper dissects Art. 5 CDSM Directive unveiling three layers of analysis. First, the substance, logic, and promises of the new provision are introduced. Second, it identifies diverging national implementation models, developing a comparative study of the Hungarian, German, and Italian experiences. Third, drawing from the comparative study, the paper provides fundamental guidance in understanding whether Art. 5 CDSM Directive can be considered an evolution or, rather, a devolution of the copyright teaching exceptions in Europe. Focusing on the systematic concerns arising from the new provision, its embedded limits, and the strategic uses of the licensing carve-out, we conclude that the EU legislature has only partially achieved the goal of striking a more European, modern, and sustainable balance between copyright protection and the right to education in the digital environment.

## Introduction

In 1709, the Statute of Anne, the first copyright statute in the world, declaimed “the encouragement of learning” as the *raison d’être* of copyright in its original form.^1^ Alongside the need to dignify and protect writers from the threat of misery, the Statute explicitly aimed at valorizing knowledge and facilitating access to “useful books”^2^ in the interest of society. The private and public objectives pursued by the Statute were designed as a harmonious coexistence of purposes, which however turned out to become the key dilemma of modern and, in particular, digital copyright law.^3^ One of the facets of this dilemma is represented by the complicated relationship between copyright and education. Over the centuries, the consolidation and expansion of the protection of copyright’s exclusive rights at the international, supranational, and national levels has relegated the possibilities for educators and students to use works and other subject matter protected by copyright to the sparse shadows of a fragmented landscape of exceptions and limitations. Especially in a digital and, by default, borderless environment, this contrast in regulatory attention has created widespread uncertainty, or rather resulted in the certainty that “not using” is the safer option. In the absence of strong imperatives in form and substance to include an exception for teaching purposes in their national laws, the Member States of the EU have created a diverse landscape of copyright exceptions and limitations for educational uses of protected works, which resembles more of a missed opportunity^4^ rather than evoking the solemn promise of Queen Anne’s “Act for the Encouragement of Learning”.

Under the aegis of the Berne and the Rome Conventions, which allow the contracting parties to derogate from the protection of copyright and related rights, respectively, to permit *ex lege* certain uses of works for teaching purposes,^5^ the EU Member States each developed their own legal understandings and regulations on the teaching exceptions contained in the international instruments, with largely varying outcomes.^6^

Since 2001, in an attempt to harmonize the landscape of exceptions and limitations in the EU, the cornerstone of permitted educational uses of works protected by copyright has been Art. 5(3)(a) InfoSoc Directive.^7^ Usually referred to as the “illustration for teaching exception”, this provision allows national legislators to introduce exceptions or limitations permitting the reproduction and communication of protected content for education and scientific research, to the extent justified by their non-commercial purpose and as long as the source and author’s name are indicated. This first attempt by the EU legislators to harmonize national provisions on the use of works and other subject matter protected by copyright for teaching purposes suffers from the often-lamented defects and shortcomings of the list of copyright exceptions set in Art. 5 InfoSoc Directive, namely, *in primis*, its exhaustive and optional nature.^8^

Unsurprisingly, Art. 5(3)(a) InfoSoc Directive has been transposed disharmoniously across the EU Member States.^9^ Within the boundaries drawn by the exception, i.e. that any use of relevant material for the sole purpose of teaching indicates the source and the author’s name and does not go beyond the extent justified by the non-commercial nature of the use, Member States have developed a rich variety of legal approaches. The spectrum ranges from restrictive legislative designs, which for instance authorize the use of only parts, short fragments, or a limited number of works,^10^ to highly specific legal approaches that refer to the illustration for teaching as well as to permitted uses for school broadcasts and school media collections,^11^ up to broader and more flexible legal wording enabling uses of protected materials within the boundaries of the educational scope.^12^ Another diverging element in the national implementations of the InfoSoc Directive’s “illustration for teaching” exception is the Member States’ decision on whether to foresee a fair compensation to be paid to the right holder. Numerous EU Member States opted for various mechanisms to guarantee the payment of a fair remuneration to the copyright holders.^13^

The discrepancies across the EU regarding how to strike a balance between strong copyright protection on the one hand and the right to education on the other hand, which received concrete expression in the provision of the international instruments and in Art. 5(3)(a) InfoSoc Directive, make it evident that fragmented national solutions hardly meet the needs of the evolving and ever more digital learning environments. The unsatisfactory nature of the EU copyright norms regarding teaching activities has become even more prevalent with the breakout of the SARS-CoV-2 (COVID-19) pandemic in Europe in early 2020. In almost every EU Member State, governments decided to shut down primary, secondary, vocational, and higher education institutions and replace in-person education with remote solutions, causing significant legal uncertainty.^14^ This has inevitably exacerbated the need for technology-proof and cross-border legal provisions in the EU that can effectively strike a balance of interests in regular times as well as during public emergencies.^15^

Discussions among EU policymakers concerning this need started way before the outbreak of the pandemic. To overcome the shortcomings of Art. 5(3)(a) InfoSoc Directive and the resulting fragmentation of national legal regulations on the teaching exception, the possibility of a stronger harmonization was proposed in 2014 along with the first draft of the Directive on Copyright and Related Rights in the Digital Single Market (CDSM Directive).^16^ In what has been described as an epochal transition in the EU harmonization of copyright exceptions,^17^ Art. 5 CDSM Directive introduces a new exception tailored to the uses of works and other subject matter in digital and cross-border teaching activities.^18^ The main goal of this analysis is, first, to contextualize and dissect the building blocks of this new provision, calling for attention to the impact of a new digital teaching exception in the EU (Sect. 2). Second, the paper will examine some of the implementation models that are arising across the Member States (Sect. 3). Third and last, the paper critically evaluates the newly introduced regime under Art. 5 (Sect. 4), leading to insightful conclusions on its role and impact in the ongoing process of reform and modernization of copyright rules in the EU.

## The New Digital Teaching Exception or Limitation

The first proposed draft of the CDSM Directive provides some insights into the rationale behind the introduction of a new cross-border teaching exception solely for digital uses. The explanatory memorandum to the draft^19^ considered three options to address the shortcomings of Art. 5(3)(a) InfoSoc Directive. Under the first option, the European Commission considered providing guidance to the Member States on the application of the existing teaching exception^20^ and organizing a stakeholder dialogue for that purpose. This option was considered insufficient as it would have failed to provide legal certainty, in particular as regards cross-border uses. Although not expressly addressed in the explanatory memorandum, the optional nature of the teaching exception set in the InfoSoc Directive would most likely have created the most severe obstacle with additional effects on the cross-border use due to fragmented national implementations. Accordingly, the second option was to introduce a mandatory exception “with a cross-border effect covering digital uses”. A third option included the second option with the added possibility for Member States to make the application of the exception dependent on whether licences are available. This third option was eventually adopted as the most proportionate one.

The recitals of the proposed draft and of the eventually adopted CDSM Directive provide further insights into the motivation of the EU legislators. Recitals 5 of the draft^21^ and of the final text of the CDSM Directive both stress the necessity to reassess certain exceptions and limitations in the light of technological changes and new types of uses of works and other subject matter protected by copyright that are not covered by existing exceptions or limitations. In particular, the cross-border effects of unharmonized exceptions could constitute barriers to the functioning of the internal market. More concrete guidance is provided by the recitals specifically dedicated to the new digital teaching exception.^22^ It is unsurprising that the recitals of the final text of the Directive are more elaborate and provide more specific guidance compared to those of its first proposed draft, as well as certain nuances suggesting to national legislators how this new exception should be transposed and applied.^23^

The overarching rationale of the exceptions is to provide educational establishments with legal certainty when they use protected works or other subject matter in digital teaching activities, *including* online and cross-border teaching.^24^ This implies, and it is confirmed by recital 21 CDSM Directive, that digital uses in general are covered, and not only such uses with a cross-border dimension.^25^ However, Member States may determine the extent to which specific material can be used. Recital 21 CDSM Directive underlines that “[i]n most cases … only parts of extracts of works” can be used to fulfil the purpose of illustration. The reproduction, communication or making available to the public of material should not serve as a substitute for the purchase of teaching materials. These concrete limitations to the substantive scope were only introduced after the first proposed draft of the Directive and indicate the intention of the EU legislators to interpret the scope of the Directive narrowly, in line with the three-step test.^26^ The narrow scope of the exception is further illustrated by recital 22 CDSM Directive, which was also only introduced after the first proposed draft of the Directive. Although it generously defines the scope of the activities of an educational establishment, uses of protected material “should be limited to what is necessary to the purpose of such activities”.

### A Basic Mandatory Exception...

The teaching exception introduced by Art. 5 CDSM Directive differs significantly from that of Art. 5(3)(a) InfoSoc Directive. The exception of the latter permits the “use for the sole purpose of illustration for teaching or scientific research, as long as the source, including the author’s name, is indicated, unless this turns out to be impossible and to the extent justified by the non-commercial purpose to be achieved” in relation to the exclusive rights of reproduction (Art. 2 InfoSoc Directive) and communication to the public and making available (Art. 3 InfoSoc Directive).^27^ Article 5 CDSM Directive stretches the scope of the digital teaching exception also to the rights contained in the CDSM Directive itself,^28^ and certain rights of the Database Directive^29^ as well as of the Computer Programs Directive,^30^ naturally excluding the right of distribution.^31^ Article 5 CDSM Directive is more restricted in that it only applies to digital uses “under the responsibility of educational establishments”. The Directive does not provide for an autonomous definition of “educational establishment”,^32^ yet clarifies that such uses must take place either on the educational establishment’s premises or at other venues, or through secure electronic environments to which only teaching staff and students have access.^33^

Unlike the InfoSoc teaching exception, Art. 5 CDSM Directive is obligatory, meaning that Member States must implement the exception into their national laws. However, Art. 5(2) CDSM Directive contains an optional carve-out to the mandatory first paragraph, or, in other words, an *exception to the exception*. This option permits Member States to restrict the application of the exceptions to cases in which no appropriate licences are available. The Directive formulates this differently by providing that Member States can foresee that the exception “does not apply or does not apply as regards specific uses or types of works or other subject matter … to the extent that suitable licences … are easily available on the market”. For that purpose, Member States must ensure that the relevant licences are “available and visible”. In addition, or alternatively, Member States can make the application of the exception subject to mandatory remuneration.

### … with a Prominent Licensing Carve-Out

The remarkable harmonizing effect of Art. 5 CDSM Directive is partially diminished by the licensing carve-out, which allows Member States to give priority to licensing schemes stemming either from the implementation of Art. 5(3)(a) InfoSoc Directive or newly introduced in a broader fashion with the transposition of Art. 5 CDSM Directive.^34^ The carve-out can be tailored to the specific needs of the implementing Member States, suggesting that licences can cover either all or only “specific uses or types of works or other subject matter”.^35^ The provision is clear, and recital 23 further clarifies that uses for which licences are not available should remain free under the exception of Art. 5(1) CDSM Directive, a clarification that was not included in the recitals of the first proposed draft.

However, educational establishments should not incur a high administrative burden or be faced with legal uncertainty in relation to the use of protected works and subject matter. Therefore, if the carve-out is implemented, Member States must ensure that the licences are available and visible “in an appropriate manner for educational establishments”.^36^ This creates an obligation for Member States to take measures to ensure that educational establishments can access the relevant licences. Recital 23 further clarifies that such licences must “meet the needs of educational establishments” and that one way to ensure the visibility and accessibility of licences is to develop information tools.^37^ To ensure legal certainty, “Member States should specify under which conditions an educational establishment can use protected works or other subject matter under that exception and, conversely, when it should act under a licensing scheme”.^38^ Although the general objective to reduce the burden on Member States was already included in the first proposed draft,^39^ the Directive in its final version provides more guidance on the concrete implementation of this obligation, suggesting, for instance, that Member States could require the permitted uses for digital teaching activities to respect the moral rights of authors and performers.^40^

### The National Implementation Blueprint

Article 5 contains a mandatory core and some elements that allow Member States to fine-tune their domestic version of the teaching exception. To begin with, it is notable that, as opposed to Art. 5(3)(a) InfoSoc Directive, which provided for an exception for teaching and research, Art. 5 CDSM Directive applies exclusively to digital and cross-border *teaching* activities. This means that, since 7 June 2021,^41^ if not already foreseen, Member States must feature in their national laws a teaching exception that covers, *at least*, digital teaching activities.

It is now mandatory that national transpositions include the following four conditions. First, the digital teaching exception shall include the country-of-origin clause to enable a cross-border use of works and other subject matter. Second, the permitted digital uses must take place under the responsibility of an educational establishment and in a “secure digital environment”.^42^ This has the consequence that Member States shall expressly indicate, and at some point define,^43^ the following criteria: (1) non-commercial,^44^ (2) educational establishments,^45^ (3) premises, and/or (4) secure digital learning environment. Third, in order to ensure the effectiveness of the provision, Member States must foresee that the exception cannot be overridden by contract.^46^ This will, of course, not apply to the contractual override expressly permitted by way of the licence carve-out. Fourth and last, the national digital teaching exception must include the obligation of indicating the author and the source of the used material.

Once these mandatory elements are included in the transposition, Member States are left discretion to make two decisions that decisively conclude the process of legislative drafting: the application of a fair compensation mechanism^47^ and of the licensing carve-out. From these two crucial choices, which are not mutually exclusive, stem four possible options:

1. All uses are permitted and subject to statutory compensation;
2. All uses are permitted with no fair compensation;
3. Only some uses (or uses of only some works) are permitted and subject to statutory compensation;
4. Only some uses (or uses of only some works) are permitted with no fair compensation.

## Emerging National Implementation Models of Art. 5 CDSM Directive

The analysis focuses on a selection of three Member States. Besides being fairly representative of the geographical and cultural diversity that the EU enshrines and promotes, the cases of the evolving Hungarian, German, and Italian copyright legislation showcase particularly meaningful differences in their approaches to the digital teaching exception.

The Italian case is characterized by a peculiar approach to the copyright exceptions on educational activities. On the one hand, the Italian transposition of the InfoSoc teaching exception shows a restrictive approach allowing solely for the “summary, quotation, or reproduction of parts of works” to be communicated to the public for the purpose of education or scientific research^48^ and publicly performed within the “walls” of educational institutions.^49^ On the other hand, even before the transposition of the CDSM Directive, Italian copyright law provided an additional unique exception specifically tailored to the online educational use of low-resolution images and music works without any authorization.^50^

Hungary’s pre-CDSM logic of the teaching exception could be similarly characterized as a primarily “brick-and-mortar” exception. The use of protected works and subject matter for teaching purposes was either limited to the premises of the educational institution (including, of course, the use of digital means to present materials on-the-spot); or to the sharing of tangible copies of materials (reproduced strictly in line with the number of involved students) among the participants of the educational event or examinations. Since 2009, however, Hungary’s Copyright Act includes a unique exception that allows the adaptation (including digital reuses) of works and other subject matter for educational purposes as a part of the educational exercise as well.^51^

Lastly, the remodelled German teaching exception has undergone a partially liberating evolution. While before the transposition of Art. 5 CDSM Directive certain types of works were generally excluded from the scope of the exception, after transposition their use for teaching purposes is only prohibited if suitable and appropriate licences are available. Examining how these three countries embraced the novelties of Art. 5 CDSM Directive and reformed their legal systems therefore provides an insightful overview of the impact of the provision and its prospective legal effects.

### Hungary: The Response to the State of Danger

The Hungarian Ministry of Justice, in close collaboration with the Hungarian Intellectual Property Office (HIPO), prepared the first, not publicly available version of the implementation draft by the end of the summer of 2019. In line with this draft, the Ministry of Justice and HIPO organized six preparatory public consultation meetings on key areas of the CDSM Directive. Shortly after the COVID-19 pandemic reached Hungary, the Parliament declared a state of danger. Based on this, the Government was granted the right to temporarily legislate via government decrees from as early as 30 March 2020.^52^

Education in Hungary switched from in-person to remote from 16 March 2020. In the absence of a safe copyright exception for the benefit of teachers and educational institutions to enable them to share third-party contents with students, a pressing need emerged to introduce the new digital teaching exception via a governmental decree. Such a decree was published on 16 April 2020.^53^ The implementation of Art. 5 CDSM Directive took its final form by the acceptance of Act LVIII of 2020 on 16 June 2020 on the cessation of the state of danger.^54^ This law has transposed the rules of the government decree into the national Copyright Act. In this way, Hungary became the first Member State of the EU to implement Art. 5 CDSM Directive.

The preparations for the implementation of the rest of the CDSM Directive did not stop during the pandemic. Following almost a full year of drafting and consultations, the Parliament passed Act XXXVII of 2021 on 28 April 2021.^55^ The transposition of Art. 5 CDSM Directive thus occurred in two main phases. Act LVIII of 2020 amended several existing articles of the Copyright Act and introduced several other articles. In the second, more formal phase, Act XXXVII of 2021 renumbered and amended a few of the relevant articles.

The key novelties of the reform are as follows. Article 33/A introduces the definition of secure electronic systems. Article 34(3a) codifies the country-of-origin approach by declaring that the relevant use is deemed to occur on the soil of the country where the educational institution is domiciled. The new exception allows for the on-the-spot digital and online educational use of works that “borrow” from third parties’ works or other protected subject matter (“grand citation”);^56^ the making and presenting of derivative works (adaptations) in the course of (in-person, synchronous) digital and distance education;^57^ as well as for distributing and making available to the public via secure electronic systems parts of books or (full) journal or newspaper articles for purposes of education or examination, in line with the number of involved students.^58^ These provisions represent a continuity in the logic of the Hungarian copyright system. All provisions are either verbatim implementations of the CDSM Directive’s provisions or “digital updates” to the formerly existing educational exceptions. This is not to say that the new rules are meaningless. Indeed, they effectively clarify the extended scope of lecturers’ and students’ possibilities in the digital educational environment.

The Hungarian legislation, however, introduced a unique “teaching-like” exception in Chapter X of the Copyright Act focusing on certain specific works. According to Art. 68(2), the use of the image of works of fine art, architecture, applied art, industrial works of design which constitute an artistic creation and photographic works shall be allowed in the course of scientific dissemination of knowledge. This rule replaced a prohibition of quoting from the subject matter listed above. Codifying special rules – including exceptions – in special chapters of the Copyright Act has its own history in Hungary (and certainly elsewhere, too). Hence, limiting the scope of an exception to certain works poses no general problems, especially as the general (educational) exceptions remain applicable to works of fine art, architecture, applied art, industrial works of design which constitute an artistic creation and photographic works. Yet what happened here seems to represent the introduction of a completely new exception that is only “teaching-like” (expressly called “scientific dissemination of knowledge” or “*tudományos ismeretterjesztés*”). It is clearly outside the scope of institutional education and hence also applies to users that are not covered by the concept of educational institutions. Therefore, it is not impossible that this article of the Copyright Act will be tested in front of courts.

### Germany: Opening Up Via the Licensing Carve-Out

The German Ministry of Justice and Consumer Protection published its first discussion draft on 15 January 2020 and a little more than a year later the Government released its own draft on 3 February 2021. After a first reading in the German Parliament, the draft law was discussed in the relevant committees and passed the second and third readings on 20 May 2021, and was formally adopted on 31 May 2021. The law for the adaptation of copyright to the requirements of the digital single market was published in the official journal on 4 June 2021 and came into force on 7 June 2021.

Germany transposed Art. 5 CDSM Directive on 31 May 2021 along with all other provisions of the Directive in the Act to Adapt Copyright Law to the Requirements of the Digital Single Market.^59^ While most other provisions of the CDSM Directive were transposed into the German Act on Copyright and Related Rights, Art. 17 was given its own separate law. The German Copyright Act already included an exception for teaching in educational establishments in Sec. 60a. The provision itself was already technologically neutral before its adoption following the implementation of the CDSM Directive into German law.

The exception in Sec. 60a was only adapted in two regards. First, it introduced a licensing option for certain uses and, second, it implemented the country-of-origin approach mandated by Art. 5(3) CDSM Directive. To understand the effect of these changes, it is necessary to examine the new provisions in their entirety. In particular, the inclusion of a licensing option in Sec. 60a(3), second sentence, seems to suggest that the exception became more restrictive and limited education establishments and their members in using works and other subject matter protected by copyright. Due to the construction of Sec. 60a in its pre-transposition version, this is arguably not entirely accurate.

Starting with the general rule, the exception permits the use of up to 15 percent of published works for the purpose of illustration in teaching in educational establishments for non-commercial purposes.^60^ Certain types of works, such as illustrations, individual articles from scientific journals and out-of-commerce works can be reproduced in their entirety.^61^

The teaching exception does not apply to certain uses of works, which are exhaustively listed in Sec. 60a(3), including works that are “exclusively suitable, intended and labelled for teaching in schools”. This is where the newly introduced licensing carve-out arguably produces beneficial effects. While under the old version of Sec. 60a the uses expressly listed were excluded from the scope of the exception, such uses now become permitted under certain conditions. A second sentence newly included in the third subparagraph states that the general prohibition of certain uses shall only apply when licences for such uses are easily available on the market. If such licences are not available, the first sentence of Sec. 60a(3) does not apply and the exception to the exception does not create any effects. The result of this mechanism is that right holders or their representatives can determine whether the exception is applicable without conditions or whether the uses listed under the exception to the exception require a licence.

Even prior to the adoption of the CDSM Directive, German copyright law made the application of the teaching exception subject to compensation, with certain exceptions.^62^ This has not changed after the Directive’s transposition.

### Italy: Restricting Access Via the Licensing Carve-Out

The Italian process of implementation of the CDSM Directive was concluded on 4 November 2021. Despite the fact that Italy was among the first European countries to be hit by the COVID-19 pandemic in March 2020, the implementation of Art. 5 was not prioritized via a fast-track procedure in order to facilitate the transition to online teaching across the country, as opposed to what happened in Hungary.

The Italian Parliament took the first step to implement the Directive on 20 April 2021 when it approved Act No. 53 of 22 April 2021, also known as the 2019–2020 European Delegation Law. It thereby officially mandated the Government to transpose, among others, the CDSM Directive into national law and determined the guiding principles that had to be followed. In Art. 9 of the European Delegation Law, the Parliament explicitly instructed the Government to “exercise the option set in Article 5(2) [CDSM Directive], which allows to exclude or limit the application of the exception or limitation ex paragraph (1) of the same Article for specific uses or types of works or other subject matter”.^63^

Following these indications, the Italian Government promptly prepared a first draft of the implementation decree, which was not made publicly available, yet was shared with and commented on by the National Permanent Advisory Committee on Copyright Law.^64^ Article 4 of the draft suggested a systematic reform of the existing copyright exception for quotation and teaching uses (Art. 70 Italian Copyright Act), expanding its scope beyond “literary and artistic works” also to “other subject matter” and explicitly including digital teaching activities. The draft also proposed to generously exercise the licensing carve-out enshrined in Art. 5(3) CDSM Directive excluding sheet music and “all those cases when” (“*tutte le ipotesi in cui*”) an appropriate and reasonable licence was easily available on the market. No fair compensation was foreseen, thus leaving this particular aspect of the existing teaching exception under Art. 70 intact.

After several stakeholder dialogue sessions, on 6 August 2021, the Government transmitted a consolidated draft of the implementation decree to both Chambers of Parliament,^65^ which eventually expressed positive opinions on it, thus leading to its official adoption and publication in the official gazette on 27 November 2021.^66^ As a result, the Italian transposition of Art. 5 CDSM Directive significantly changed from the draft initially proposed by the Government. Instead of taking the opportunity to reform, re-systematize, and modernize the existing teaching exception set in Art. 70 Italian Copyright Act – similarly to what Hungary and Germany did – the implementation decree has now introduced an additional provision specifically regulating uses for digital teaching activities.

The new article, Art. 70^bis^ Italian Copyright Act, permits the “summary, quotation, reproduction, translation, adaptation, and communication to the public” of parts of works and other subject matter by way of digital means for non-commercial teaching purposes, with no fair compensation involved. This new exception does not apply to sheet music and educational materials for which a voluntary licence is easily available on the market. Thus, compared to the very first proposal by the Government, the boundaries of the licensing carve-out have shrunk to these two categories of works only.

Even though this may look like a development towards a more permissive approach, this is mostly a mirage. In fact, the introduction of the new digital teaching exception in the Italian copyright system represents a tighter grip on the access and use of protected works by teachers and students. Under the pre-existing legal framework, they could use parts of *any* work within the extent necessary for their non-commercial teaching purposes. Additionally, Art. 70(1^bis^) enabled them to use online low-quality images and musical works for their educational activities. Lacking an adequate systematization of such provisions, the new Art. 70^bis^ precludes the possibility of using parts of educational materials (e.g. textbooks, handbooks, anthologies) in online learning environments, as it imposes an unprecedented favouring of voluntary licences. Highly uncertain remain the effects on images and musical works, as the hierarchical relationship between the “low-quality” requirement set in Art. 70(1^bis^) and their potential “educational” nature under Art. 70^bis^ remains unspoken.

## The EU Digital Teaching Exception: Evolution or Devolution?

### Systematic Concerns

As seen above, the CDSM Directive intended to mitigate the concerns raised by Art. 5(3)(a) InfoSoc Directive and to create the conditions for digital teaching fit for the 21st century. The reference to digital technology, the cross-border nature of the exception or limitation, and the obligatory nature of the new provision, however, cannot conceal the new, systematic concerns stemming from Art. 5 CDSM Directive.

First, the new rules introduced by Art. 5 are narrow in scope.^67^ It is not doubted that the current limits to the scope of Art. 5 CDSM Directive guarantee that the teaching exception complies with the three-step test.^68^ More complicated to address is the question of the potential *lex specialis* nature of the provision in relation to Art. 5(3)(a) InfoSoc Directive. Two elements seem to lead towards a rejection of such labelling: on the one hand, the absence of a mandatory general teaching exception at EU level, and, on the other hand, the heterogeneous implementations of Art. 5(3)(a) InfoSoc Directive at national level.^69^ In other words, in the absence of a *lex generalis*, i.e. a fully harmonized general teaching exception implemented across all EU Member States, there can be no *lex specialis*. The narrow scope of Art. 5 CDSM Directive is further exemplified by the limited scope of the notion of “education”. Since the activities referred to in the provision are limited to formal and institutional teaching and learning experiences, other forms of knowledge-sharing activities that take place outside the framework of “educational establishments” are excluded from the scope of digital education. This also includes activities carried out by civil society and non-governmental organizations, or extra-curricular teaching and learning activities promoted by educators outside of institutional facilities. However, such activities, as long as they are performed for non-commercial purposes, could fall under a general teaching exception, if and where adequately implemented.

Second, Art. 5 CDSM Directive leaves plenty of flexibility for Member States to adjust the digital education exception or limitation to their specific needs. While, on the one hand, this is absolutely in line with the logic of harmonization by means of Directives, on the other hand, such flexibilities have turned out to be an encumbrance of the uniform implementation and interpretation of the rules of the InfoSoc Directive. As shown above, the three analyzed countries follow three far from uniform solutions to the same legal challenge. Consequently, one of the major goals of the CDSM Directive, to enhance the effectiveness of the rules on digital education in the EU, cannot be completely achieved.

Third, Art. 5 CDSM Directive cannot be viewed in isolation. Other parts of the CDSM Directive, as well as of the InfoSoc Directive, overlap with the new digital teaching exception. For example, Art. 6 CDSM Directive allows for new (digital) exceptions for the benefit of libraries, which are quite often connected to educational institutions.^70^ Furthermore, Art. 5(2)(b) InfoSoc Directive on the private copying exception also partially overlaps with the new teaching exception in that it applies to the same subject exercising a particular activity: students accessing learning materials.^71^ In certain situations, for example in the case of higher education, multiple limitations and exceptions come into play at the same time. Among them, university libraries are allowed to preserve full copies of works that lecturers can only partially share with their students (even if free of charge), from which students may only create copies to an even more limited degree and against payment of private copying levies, and the ultimate goal of accessing knowledge for educational purposes is limited. We could reasonably conclude that Art. 5 CDSM Directive did undoubtedly positively address certain inconsistencies of the copyright law of the EU, but has similarly opened a Pandora’s box that has released a number of other curses.

### Quantity of the Work

As highlighted from studies on the pre-CDSM legal landscape,^72^ the determination of the amount that teachers and students are permitted to use within the scope of educational activities is pivotal to determining the scope of any teaching exception. Despite the ubiquitous presence of the three-step test, limiting the application of any copyright exception or limitation to “certain special cases which do not conflict with a normal exploitation of the work or other subject-matter and do not unreasonably prejudice the legitimate interests of the right holder”,^73^ Art. 5 CDSM Directive allows for a margin of appreciation by Member States to determine whether full works or only parts thereof can be freely used by the targeted beneficiaries.

All three countries under analysis opted to specify the amount of a work that is permitted to be used for digital teaching activities – yet all adopted different strategies. The new Hungarian digital teaching exception allows for the use of *parts* of books or *full* journal or newspaper articles. In this case, the emphasis on the limited amount is not on the excerpt of the work, but rather on the number of copies that can be reproduced and made available, which must be in line with the number of students involved. The Hungarian legislature also introduced an additional provision clarifying that the use of the image of works of fine art, architecture, applied art, industrial works of design which constitute an artistic creation and photographic works shall be allowed (in full) in the course of scientific dissemination of knowledge.

The Italian scenario is slightly different. According to the new digital teaching exception, the “summary, quotation, reproduction, translation, and adaptation” of only *parts* of works and other subject matter is allowed. From a literal reading of the provision, it could be open for discussion whether the full works can be communicated to the public.^74^ However, the legislative text closely resembles the wording of the pre-existing teaching exception, which allows for only “fragments” of the works to be communicated to the public for teaching purposes.^75^ Worth noting is that no specific indication was provided by the Italian legislature with regard to the use of artworks, works of visual art or photography, thus making it possible for a strict interpretation of the provision to apply only to “parts” of such works.

Lastly, Germany presents a particularly interesting scenario, as already in the original (pre-CDSM implementation) teaching exception the use of published works for the purpose of illustration for teaching was limited to 15 percent of the work.^76^ The indication of a precise percentage is an atypical legislative design across the EU copyright landscape and represents a distinctive feature of the German teaching exception, which the legislators also preserved in the implementation of the digital teaching exception. Some argue that this represents a missed opportunity for the German copyright legal system to undertake long overdue steps towards an increase of such quantity and an enhanced flexibility in the educational uses.^77^ However, similarly to what occurred in Hungary, certain types of works, such as illustrations, individual articles from scientific journals and out-of-commerce works, can be reproduced in their entirety.^78^

### Strategic Use of the Licensing Carve-Out

The purpose of the licensing carve-out was to enable Member States to maintain existing licensing arrangements that were already in place at the time of the adoption of the CDSM Directive.^79^ Before the adoption of the Directive, Member States were free not to adopt an exception that would enable unauthorized uses for the purpose of illustration for teaching, and lawful uses had to rely on some sort of a licensing system. Indeed, such systems were in place in some Member States, especially in the Nordic countries.^80^

Article 5 CDSM Directive offered Member States a variety of options in which form to introduce a new teaching exception or in which way to restructure their existing exceptions. Whether to enable the override of the basic exception with the possibility for right holders to offer suitable licences constitutes an opportunity to fine-tune the exception to appease domestic publishing markets. This is the sense in which recital 23 must be understood when it refers to “[d]ifferent arrangements ... to facilitate educational uses of works and other subject matter” which are already in place in Member States. To avoid disturbing these established mechanisms, and to ensure remuneration for publishers in parallel to a generally applicable exception, Member States can limit the licensing carve-out for specific types of works and uses. Such arrangements have usually been developed by taking account of the needs of educational establishments and of different levels of education. This construction anticipates that for certain works or subject matter a licensing market does not and most likely will not exist, but that for specific types of subject matter, for example textbooks and sheet music, licensing income constitutes an important revenue stream for publishers.

Member States can intervene more or less intensively in the market mechanisms by restricting the licensing carve-out to specific works, and possibly encourage the availability of licences in relation to the relevant category of works. In this context, Member States, which choose to implement Art. 5(2) in their domestic laws, also incur an obligation “to ensure that the licences authorising the acts [within the scope of the exception of Art. 5(1)] are available and visible in an appropriate manner for educational establishments”.

Against this background, Hungary takes the most extreme position by implementing a “pure” exception which does not permit the override by licences. Any licence offered would then also violate Art. 7(1) CDSM Directive which prohibits contractual overrides of the exceptions contained in Arts. 3, 5 and 6 CDSM Directive. Italy, on the other hand, makes generous use of the licensing carve-out and permits a contractual override within the scope of the digital teaching exception for sheet music and educational materials. Germany took a middle course by excluding certain specific acts and types of subject matter from the application of the generally applicable exception under Sec. 60a(1) German Copyright Act as long as licences for such uses are easily available and accessible. What is special about the German implementation is that it turned a derogation of a general teaching exception into uses that require authorization only if appropriate authorization mechanisms (suitable and easily available licences) exist.

Making these licences work is as much a task of the right holders as it is a task of the Member States. They expressly incur an obligation to ensure the availability and visibility of such licences. Effectively, licences that are difficult to identify or to obtain will fall short of this standard and would therefore not result in an override of the general teaching exception.

Finally, independently or in combination with a licensing carve-out, Member States can choose to make the digital teaching exception subject to fair compensation, or they might decide to act like Hungary and Italy by opting for no compensation in this regard.^81^ Of course, a combined model, such as the German one, must take into consideration that licensed uses are not subject to additional statutory remuneration.^82^ In determining fair compensation, Member States are bound by the jurisprudence of the ECJ which has developed fair compensation into an autonomous concept of EU law.^83^

### Assessing the “Suitability” of Licences

Article 5(2) CDSM Directive further requires that the inapplicability of the general exception under Art. 5(1) is subject to the availability of “suitable licences authorising the acts referred to in paragraph 1” of Art. 5 and that such licences cover “the needs and specificities of educational establishments [and] are easily available on the market”. Recital 23 merely states that “licensing schemes should meet the needs of educational establishments”, without specifying what these needs are and what, as a result, would make licences suitable.

These are, however, aspects that are crucial for licensing models to work. Suitable licences would certainly be easily accessible and visible for educational institutions, or, in other words, institutions benefitting from the exception under Art. 5 should have no difficulties in identifying relevant licences. Ideally, licensing offers would be made available through one or a small number of contact points or managed by the relevant collecting societies. In any case, transaction costs should be kept low so that even small or medium-sized educational institutions would be able to shoulder the administrative burden of obtaining licences for the content used by their teachers.

The flexibility offered by the CDSM Directive here is necessary, considering the organizational models of primary, secondary, and higher education in the various Member States. While Germany’s educational sector is organized in a decentralized manner with the federal states having competence to determine curricula at primary and secondary school level, the Hungarian and Italian primary and secondary education systems are centrally organized. Such centralized systems are likely to have a more direct influence over the materials teachers can choose from, even though margins for the individual creativity of educators are to be expected. Universities enjoy a particularly high degree of independence by virtue of the principle of academic freedom, where lecturers at this level of education might be able to rely on Art. 5 with greater success and efficiency.

Neither the Italian nor the German implementations specify the terms “suitable” licences “easily available on the market”, which transpose the last sentence of Art. 5(2) CDSM Directive. Furthermore, the notion of suitability has a substantive dimension. Accordingly, licences must be suitable for the purposes of educational establishments. Their scope, if offered, should cover the material necessary to fulfil the specific needs of educational establishments. The Directive itself provides that licences can be offered for certain specific uses or types of works or other subject matter, such as material that is primarily intended for the educational market or sheet music. It can therefore certainly be expected from institutions responsible for ensuring that licences are obtained for works or other subject matter usually made available to their students by their teachers for educational purposes by way of use in electronic environments that they inquire with relevant publishers what, if any, licences are available. But suitability must also be understood in the sense that a fragmented licence market, which would require such institutions to obtain a large number of licences covering small repertoires, is not a market that offers suitable licences. This is where collecting societies have a crucial role to play to ensure that larger repertoires are offered by way of dedicated licences to educational establishments.

The suitability of a licensing scheme that permits the use of works or other subject matter protected by copyright for educational establishments must be measured against other additional factors.^84^ Arguably, the notion of suitability must be interpreted to include an economic factor: licences cannot be overly expensive for the licensee, either individually or cumulatively. But this will depend largely on the size of institutions or whether the responsibility lies with the federal states, as in the case of Germany, or with ministerial or administrative bodies that still need to be identified, like in Italy.

## Conclusion

The EU’s attempt to strike a modern, more sustainable balance between copyright protection and the right to education in the digital environment is a long overdue ambition. By and large, Art. 5 CDSM Directive can be said to have only partially achieved this goal. The implementation of the provision in Italy, Hungary, and Germany reflects different traditions in the relationship between the state and the education sector. The main aim of the new teaching exception to enable digital uses of works and other subject matter has undoubtedly been achieved in all Member States, but this is the absolute baseline of harmonization. In other respects, the implementation models could not be more different. This, of course, raises the question whether harmonization has been achieved at all, or whether the minimalist approach, which gave the Member States a large margin of discretion, still served its purpose. The largest room for flexibility – whether to opt for a licensing carve-out or not – has been used to design different models, which in themselves might create problems at the national level. However, the country-of-origin approach will largely dispense with concerns that different implementations will have on the cross-border use of works for teaching purposes.

The problems lie at the domestic level, and will most likely only emerge in the future. One problem, as exemplified by the Italian example, is the failure to clarify the relationship between pre-existing teaching exceptions and the new digital teaching exceptions. It is largely incomprehensible why certain uses for the purpose of illustration for teaching, within quantitative limits which are quantitatively more or less precisely prescribed, should be allowed online but not offline, or the other way around. In a modern educational setting, with blended teaching and learning, it should simply not matter whether access to learning materials is made in analogue or digital form. The fears of publishers that a piracy-market of teaching materials will be created is difficult to prove. Reasonable limitations on the amount of a work that can be used within the scope of Art. 5 in either the Infosoc or the CDSM Directives is an expression of the three-step test and serves to avoid a displacement of lawful offers by pirate copies of textbooks and journal articles. In any case, Member States can make use of a remuneration requirement to mitigate the harm caused by the use of protected works in and by educational establishments.

Another critical aspect of the implementation of Art. 5 CDSM Directive is how Member States that chose to opt for the carve-out will operate a licensing framework that will serve its purpose. The vaguely formulated conditions that apply to the replacement of free uses under a (possibly remunerated) exception by a licensing model are crucial. It is important to determine what “appropriate” and “easy to find” mean. The success of these models will depend on how educational establishments or the ministries responsible for education and culture will have to operate on the licensing market. Will licences be obtained to enable teachers to make use of a variety of content, for example to provide a variety of opinions and background on reading lists for advanced seminars? Will easily available and affordable licences determine what textbooks will be used at secondary level, or will textbooks and other material be selected based on their quality and pedagogical value? And will small publishers, whose licences are not so attractive in scope and in price to compete successfully with larger publishers, suffer? While these questions cannot be answered globally, it is also too early to judge the efficiency of national implementations at this point. But it is more than speculation to assume that the modalities under which licences will be made available to educational institutions or other institutional actors will play an important role in the success of national implementations. In the end, it is not only important that teaching and learning environments can rely on some teaching materials, but that a variety of materials is available in forms and access modes that can reflect and promote the political and cultural diversity of a Europe that lives and teaches unity in diversity.

## Footnotes

1 An Act for the Encouragement of Learning, by Vesting the Copies of Printed Books in the Authors or Purchasers of such Copies, During the Times therein mentioned (1710) (Statute of Anne), available at: Lionel Bently and Martin Kretschmer (eds.), “Primary Sources on Copyright (1450–1900)”, <www.copyrighthistory.org>.

2 *Ibid*.

3 *See* Cornish (2010), pp. 21–22.

4 *See also* Xalabarder (2009): “Both the [Berne Convention] and [the EU InfoSoc Directive] provide for flexible and technology neutral exceptions for teaching purposes, which may exempt any use done as part of the instruction, as well as teaching anthologies, in any formats (analog or digital), thus clearly intended to cover face-to-face, distance and on–line teaching. However, most national legislators fail to fully take advantage of such opportunity”.

5 Art. 10(2) Berne Convention for the Protection of Literary and Artistic Works of 9 September 1886 as last amended on 28 September 1979 (Berne Convention); Art. 15(1)(d) Rome Convention for the Protection of Performers, Producers of Phonograms and Broadcasting Organizations of 26 October 1961 (Rome Convention).

6 The European Commission itself stressed that “[t]he EU exception on illustration in teaching is a good example of how Member States implement exceptions in different ways, particularly when we look at Member States’ understanding of how the exception should apply in digital environments. These differences could act as a brake on education trends like online courses, in-classroom use of technologies and digital materials and cross-border learning which have gained considerable ground in recent years”. *See* Communication from the Commission to the European Parliament, the Council and the European Economic and Social Committee and the Committee of the Regions – Towards a modern, more European copyright framework, COM/2015/0626 final, Brussels, 9 December 2015, para. 3.

7 Art. 5(3)(a) Directive 2001/29/EC on the harmonization of certain aspects of copyright and related rights in the information society (InfoSoc Directive), OJ L 167, 22 June 2001, pp. 10–19. On the educational exception related to databases, *see* Art. 9(b) Directive 96/9/EC on the legal protection of databases (Database Directive), OJ L 77, 27 March 1996, pp. 20–28. On the educational exception related to related rights, *see* Art. 10(1) Directive 2006/115/EC on rental right and lending right and on certain rights related to copyright in the field of intellectual property (Rental Directive), OJ L 376, 27 December 2006, pp. 28–35. For a detailed analysis of these three distinct provisions, *see* Walter and von Lewinski (2010), pp. 733–735, 336, respectively.

8 *E.g*. Guibault (2010); Jütte (2017), pp. 242–245; Rendas (2021), pp. 154–165.

9 *See* Xalabarder (2007), pp. 390–395; Nobre (2017); Rendas (2021), pp. 169, 183–184; Lazarova (2021).

10 *E.g*. Art. L.122-5-3°(e) French Intellectual Property Code; German Copyright Act; Sec. 34 Hungarian Copyright Act; Art. 70 Italian Copyright Act; Sec. 57(3) Irish Copyright Act.

11 *E.g*. Secs. 47 and 60b German Copyright Act; Sec. 45 Austrian Copyright Act.

12 *E.g*. Sec. 19 Latvian Copyright Act; Secs. 44 and 45 Slovakian Copyright Act.

13 *E.g*. Secs. 42g, 45, 51, and 56c Austrian Copyright Act; Art. 85 Croatian Copyright Act; Secs. 13 and 18(1) Danish Copyright Act; Secs. 14 and 18 Finnish Copyright Act; Art. L122-5-3°(e) French Intellectual Property Code; Sec. 47 German Copyright Act; Art. 16 Dutch Copyright Act; Secs. 18 and 42c Swedish Copyright Act.

14 Only a few Member States feature express indications of digital teaching activities in their national transpositions of the InfoSoc Directive, *e.g*. Art. L122-5-3°(e) French Intellectual Property Code; Art. 22 Greek Copyright Act; Art. 70(1^bis^) Italian Copyright Act; Art. 27(2) Polish Copyright Act. *See* recital 19 CDSM Directive: “...The scope of those exceptions or limitations as they apply to digital uses is unclear. In addition, there is a lack of clarity as to whether those exceptions or limitations would apply where teaching is provided online and at a distance”. *See also* Ernst and Haeusermann (2006), highlighting the “short-sighted” approach of some Member States that prohibited the digital application of teaching exceptions at the early stages of harmonization.

15 As the European Commission has acknowledged in the Proposal of the Digital Services Act: “[t]he coronavirus crisis has shown the importance of digital technologies in all aspects of modern life. It has clearly shown the dependency of our economy and society on digital services and highlighted both the benefits and the risks stemming from the current framework for the functioning of digital services”. *See* Proposal for a Regulation of the European Parliament and of the Council on a Single Market for Digital Services (Digital Services Act) and amending Directive 2000/31/EC, COM/2020/825 final, Brussels, 15 December 2020, para. 1. *See also* Craig and Tarantino (2021); Hudson and Wragg (2020).

16 Directive (EU) 2019/790 of the European Parliament and of the Council of 17 April 2019 on copyright and related rights in the Digital Single Market and amending Directives 96/9/EC and 2001/29/EC (CDSM Directive), OJ L 130, 17 May 2019, pp. 92–125. At the international level, the only relevant development in this direction is the proposal advanced in November 2018 by civil society organizations and public interest groups to the WIPO Standing Committee on Copyright and Related Rights for a Treaty on Copyright Exceptions and Limitations on Education and Research Activities. *See* Civil Society Proposed Treaty on Copyright Exceptions and Limitation for Educational and Research Activities (TERA), available at: http://infojustice.org/wp-content/uploads/2018/11/tera11152018.pdf (last accessed 28 November 2021). The proposal has been stalling for years. Only very recently has the Committee devoted some attention to the topic in a round of stakeholder discussions. *See* WIPO Standing Committee on Copyright and Related Rights, 41st Session, available at: https://www.wipo.int/meetings/en/details.jsp?meeting_id=63929 (last accessed 28 November 2021).

17 Dusollier (2020), pp. 981–982: “[T]his Directive marks the transition of the EU intervention from mainly harmonizing existing national rules and strengthening the rights of its creative sector towards a genuine regulatory actor that purports to better organize a thriving European market and a fair society for creations, culture, and information.… Exceptions – at least some of them – have mutated from mere limitations of exclusive rights to proper enabling devices sustaining socially-benefiting uses of works and creations”.

18 *See* in general on the new exception, Jütte (2019); Despotidou (2021).

19 Proposal for a Directive of the European Parliament and of the Council on copyright in the Digital Single Market, Brussels, 14 September 2016, COM(2016) 593 final (Proposed Draft of CDSM Directive), p. 8.

20 The explanatory memorandum uses “exception” in its singular form, presumably because it only refers to Art. 5(3)(a) of the InfoSoc Directive. However, the recital, in the Proposed Draft as well as in the adopted version of the CDSM Directive, also refers to relevant exceptions contained in the Database Directive (*see* recital 19 CDSM Directive).

21 *See* Proposed Draft of CDSM Directive, p. 13.

22 Recitals 14–17 of the Proposed Draft of the CDSM Directive and recitals 19–24 of the final text of the CDSM Directive.

23 *E.g*. recital 21 CDSM Directive: “...The distribution of software allowed under that exception or limitation should be limited to digital transmission of software. In most cases, the concept of illustration would, therefore, imply the use only of parts or extracts of works, which should not substitute for the purchase of materials primarily intended for the educational market. When implementing the exception or limitation, Member States should remain free to specify, for the different types of works or other subject matter, in a balanced manner, the proportion of a work or other subject matter that can be used for the sole purpose of illustration for teaching”.

24 Recital 19 CDSM Directive.

25 Recitals 21–22 have been changed significantly in relation to their equivalent in the draft text. While recital 16 of the draft text merely states that “[t]he exception or limitation should cover both uses through digital means in the classroom and online uses through the educational establishment’s secure electronic network”, recital 22 CDSM Directive specifies that local digital uses in the classroom or in other venues on “electronic whiteboards or digital devices which might be connected to the internet” are also covered.

26 Expressly in Art. 7(2) CDSM Directive.

27 According to Art. 5(4) InfoSoc Directive, Member States may provide for an exception or limitation to the right of distribution to the extent justified by the purpose of the permitted acts of reproduction. Even though Art. 5 CDSM Directive does not explicitly refer to this possibility, Art. 25 CDSM Directive allows Member States to adopt or maintain in force broader provisions that are compatible with the InfoSoc Directive. Hence, the additional derogation from the right to distribution seems to remain a viable option in the national transpositions.

28 Most notably the new right for online uses of press publications introduced by Art. 15 CDSM Directive.

29 Art. 5(a), (b), (d), and (e) and Art. 7(1) Directive 96/9/EC of the European Parliament and of the Council of 11 March 1996 on the legal protection of databases, OJ L 77, 27 March 1996, pp. 20–28 (Database Directive).

30 Art. 4(1) Directive 2009/24/EC of the European Parliament and of the Council of 23 April 2009 on the legal protection of computer programs, OJ L 111, 5 May 2009, pp. 16–22 (Computer Programs Directive).

31 Art. 5 CDSM Directive; *cf*. recital 19 CDSM Directive.

32 *See* Art. 2 CDSM Directive.

33 Art. 5(1)(a) CDSM Directive.

34 Recital 23 CDSM Directive.

35 For example, “material that is primarily intended for the educational market or sheet music”. *Cf*. Art. 5(2) CDSM Directive.

36 Art. 5(2), second sentence, CDSM Directive.

37 Recital 23 CDSM Directive suggests collective licensing as a facilitator to avoid that educational establishments are forced to negotiate individually with right holders.

38 Recital 23 CDSM Directive.

39 Recital 17 Proposed Draft of CDSM Directive.

40 Recital 23 CDSM Directive.

41 According to the expiration of the term for transposition set in Art. 29 CDSM Directive.

42 This does not mean that the use of the new teaching exception is limited spatially to the (digital or physical) premises of educational establishments. On the contrary, it extends to activities “including during examinations or teaching activities that take place outside the premises of educational establishments, for example in a museum, library, or another cultural heritage institution”. *See* recital 22 CDSM Directive.

43 While some of these concepts might develop into autonomous concepts of EU law in the context of the CDSM Directive, it is possible that Member States will have a margin of discretion to shape concepts such as “educational establishments” against the background of their unique and culturally grown notions of public or private education.

44 Neither the organizational structure, nor the funding of the educational establishment shall play a decisive role in determining the commercial or non-commercial nature of the use of educational materials. *See* Stamatoudi and Torremans (2021), p. 692.

45 This is left undefined in the CDSM Directive. The only relevant indications are in recital 20 CDSM Directive.

46 Art. 7 CDSM Directive.

47 *Cf*. recital 24 CDSM Directive.

48 Art. 70(1) Act No. 633 of 22 April 1941 (Italian Copyright Act) (translation by the authors). The restrictive approach is buttressed by a markedly strict interpretation of the provision, as epitomized by the judgment of the Italian Supreme Court (*Corte di Cassazione*) of 7 March 1997, No. 2089. *See also* Gelato (1998).

49 Art. 15(2) Italian Copyright Act.

50 Art. 70(1^bis^) Italian Copyright Act. *See also* Margoni (2011), who emphasizes the peculiar nature of the provision and the Italian legislature’s choice of wording in referring to “free uses”, and Mansani and Del Re (2009), contextualizing and questioning the meaning of the “low quality” requirement.

51 Art. 34(4) Act LXXVI of 1999 on Copyright Law (Hungarian Copyright Act).

52 On the national implementation of Art. 5 CDSM Directive in Hungary, *see also* Mezei (2020); Keller (2020); Ujhelyi (2020).

53 Hungarian Government Decree No. 125/2020 (IV.16).

54 The law entered into force on 18 June 2020.

55 The first (full) draft bill was published on 7 May 2020 and a public consultation took place between May and June 2020. The Ministry of Justice and the HIPO, based on more than a hundred responses, published an amended draft bill at the end of July 2020. This version was offered for a targeted (semi-public) consultation in August 2020. Taking into consideration the recommendations at this stage, a third version was submitted for a final semi-public consultation in February 2021. The bill was finally submitted to the Hungarian Parliament on 31 March 2021. The Parliament passed the bill with 136 yeas, 29 nays, and 1 abstain. Act XXXVII of 2021 was published on 6 May 2021 and entered into force on 1 June 2021, a few days before the official transposition day. *See* Magyar Közlöny (2021), pp. 3184–3197.

56 Art. 34(3)(b) Hungarian Copyright Act. In Hungarian copyright law, “[a]ny use of a work in another work to a degree that exceeds quotation or citation constitutes borrowing”. At the same time, the scope of borrowing is limited to certain types of subject matter. *See* Art. 34(2) Hungarian Copyright Act.

57 Art. 34(4) Hungarian Copyright Act.

58 Art. 35(5) Hungarian Copyright Act.

59 Gesetz zur Anpassung des Urheberrechts an die Erfordernisse des digitalen Binnenmarktes, 31 May 2021, BGBl 2021 II, 1204.

60 The scope of the exception extends to teachers and students of “respective events”, teachers and examiners for uses at the respective educational establishment, and by third persons “insofar as this serves to present lessons or lectures or the results of tuition or training or learning outcomes at the educational establishment” and applies to acts of reproduction, distribution, making available to the public or otherwise communicating works to the public.

61 Sec. 60a(2) German Copyright Act.

62 Sec. 60h German Copyright Act stipulated that “communication to the public for the benefit of members of educational establishments and their families in accordance with Section 60a (1) Nos. 1 and 3 and Section 60a (2), with the exception of making the content available to the public” shall not be subject to remuneration.

63 Full translation by Priora available at: https://www.create.ac.uk/cdsm-implementation-resource-page/ (last accessed 5 December 2021).

64 Document not officially released, yet could be retrieved at: http://www.infoparlamento.it/tematiche/telecomunicazioni/schema-di-decreto-legislativo-recante-attuazione-della-direttiva-ue-2019790-del-parlamento-europeo-e-del-consiglio-del-17-aprile-2019-sul-diritto-daut.

65 https://www.camera.it/leg18/682?atto=295&tipoAtto=Atto&idLegislatura=18&tab=2#inizio.

66 The Consolidated Draft has been published with minor revisions, which address the comments raised by the Lower Chamber and Senate of the Italian Parliament. *See* Legislative Decree of 8 November 2021, No. 177 – Attuazione della direttiva (UE) 2019/790 del Parlamento europeo e del Consiglio, del 17 aprile 2019, sul diritto d’autore e sui diritti connessi nel mercato unico digitale e che modifica le direttive 96/9/CE e 2001/29/CE (21G00192) (GU Serie Generale No. 283 del 27-11-2021), available at: https://www.gazzettaufficiale.it/atto/serie_generale/caricaDettaglioAtto/originario?atto.dataPubblicazioneGazzetta=2021-11-27&atto.codiceRedazionale=21G00192&elenco30giorni=false (last accessed 28 November 2021).

67 Lazarova (2021).

68 *See* Art. 7(2) CDSM Directive.

69 Art. 25 CDSM Directive.

70 As already pointed out in relation to Art. 5(3)(a) InfoSoc Directive by Papadopoulou (2010).

71 *See* here especially Art. 5(1)(a) CDSM Directive which suggests that students are allowed to make and keep reproductions of works and other subject matter made available through secure online environments, *see also* for that purpose, recital 22.

72 *See inter alia* Nobre (2017).

73 Art. 5(5) InfoSoc Directive.

74 Depending on whether the meaning of the word “their” in the expression “their communication to the public” refers to “parts of works or other subject matter” or to the works themselves. *See* Art. 70^bis^ Italian Copyright Act (“*Sono liberi il riassunto, la citazione, la riproduzione, la traduzione e l'adattamento di brani o di parti di opere e di altri materiali e la* loro *comunicazione al pubblico se effettuati con mezzi digitali, esclusivamente per finalità illustrative ad uso didattico* …”) (emphasis added).

75 Art. 70(1) Italian Copyright Act. *See also e.g*. Italian Supreme Court (Cassazione civile sez. I), judgment of 7 March 1997, No. 2089.

76 The scope of the exception extends to teachers and students of “respective events”, teachers and examiners for uses at the respective educational establishment, and by third persons “insofar as this serves to present lessons or lectures or the results of tuition or training or learning outcomes at the educational establishment” and applies to acts of reproduction, distribution, making available to the public or otherwise communicating works to the public.

77 Hauk and Pflüger (2020), p. 383.

78 Sec. 60a(2) German Copyright Act.

79 *Cf*. recital 23 CDSM Directive.

80 *E.g*. Xalabarder (2007), p. 395.

81 Art. 5(4) CDSM Directive.

82 This is apparent from the reference in Art. 5(4) to para. 1 of Art. 5. Only such uses covered under Art. 5, *i.e*. the “pure” exception, can be made subject to fair remuneration.

83 *Cf*. Rosati (2021), Art. 5, para. 6.3, p. 125.

84 One important aspect that is reflected in the Irish pre-CDSM Copyright and Related Rights Act (Irish Copyright Act) is that the substantive scope of licences should be permissive rather than restrictive. In other words, licences should not restrict already available exceptions and override their scope. Existing exceptions, including those for teaching and research uses, should constitute the quantitative baseline of what is permitted. *See* Sec. 57C(2) Irish Copyright Act. Sec. 57(3) limits the amount that can be copied from a work to 5% of a given work within any calendar year. Another aspect of the Irish licensing model for educational uses serves to safeguard the appropriateness of licences and also serves to ensure their visibility. Any licence that derogates from the general exception found in Sec. 57 Irish Copyright Act must be certified by the Minister for Enterprise, Trade and Employment (*see* Sec. 173 Irish Copyright Act).
